# Application of *Aspergillus niger* in Practical Biotechnology of Industrial Recovery of Potato Starch By-Products and Its Flocculation Characteristics

**DOI:** 10.3390/microorganisms10091847

**Published:** 2022-09-15

**Authors:** Liang Zhang, Guangli Cao, He Liu, Zhenting Wu, Dianliang Gong, Xin Ru, Xiujie Gong, Qiuyue Pi, Qian Yang

**Affiliations:** 1 Bioengineering Center, School of Life Science and Technology, Harbin Institute of Technology, Harbin 150001, China; 2 State Key Laboratory of Urban Water Resources and Environment, Harbin Institute of Technology, Harbin 150090, China; 3 Hainan Key Laboratory of Tropical Eco-Circular Agriculture, Environmental and Plant Protection Institute, Chinese Academy of Tropical Agricultural Sciences, Haikou 571101, China; 4 Institute of Farming and Cultivation, Heilongjiang Academy of Agricultural Sciences, Harbin 150086, China

**Keywords:** potato starch by-products, recovery biotechnology, industrial scale, *Aspergillus niger*, flocculation mechanism

## Abstract

This study developed a practical recovery for potato starch by-products by *A. niger* and applied it on a plant scale to completely solve the pollution problems. Soughing to evaluate the effect of *A. niger* applied towards the production of by-products recycling and analyze the composition and characteristics of flocculating substances (FS) by *A. niger* and advance a possible flocculation mechanism for by-product conversion. After fermentation, the chemical oxygen demand (COD) removal rate, and the conversion rates of cellulose, hemicellulose, pectin, and proteins were 58.85%, 40.19%, 53.29%, 50.14%, and 37.09%, respectively. FS was predominantly composed of proteins (45.55%, *w*/*w*) and polysaccharides (28.07%, *w*/*w*), with two molecular weight distributions of 7.3792 × 10^6^ Da and 1.7741 × 10^6^ Da and temperature sensitivity. Flocculation was mainly through bridging and ionic bonding, furthermore, sweeping effects may occur during sediment. Flocculation was related to by-products conversion. However, due to severe pollution problems and resource waste, and deficiencies of existing recovery technologies, converting potato starch by-products via *A. niger* liquid fermentation merits significant consideration.

## 1. Introduction

Potato starch industry occupied above 70% of the potato processing industry in China as a processing method to maximize the economic value of potatoes. The development of the potato starch industry has brought problems of environmental pollution and waste of biomass resources, as well as strict enforcement of environmental laws, which has hindered its development. Starch production generated large wastewater quantities with high organic and high chemical oxygen demand (COD) levels that pollute the environment when discharged without appropriate treatment. It mainly includes two parts: one is the wastewater produced by extracting starch milk, which is mainly potato cell fluid with high protein content; the other is the wastewater produced in the process of extracting starch, that is, the process wastewater, mainly from the transport and washing wastewater. Potato residue is the waste material in potato starch production process with high levels of cellulose and hemicellulose but poor protein [1]. It is mainly composed of water, cell debris, residual starch particles and potato peel cells or cellular structures. Previous research on the resource utilization of potato starch by-products followed two typical pathways: one ought to satisfy discharge standards by consuming macro molecular substances in water using anaerobic and aerobic sludge bioreactors [2,3]. Although these methods effectively removed COD, continuous investment and routine sludge treatment increased production costs and led to the waste of biomass resources. The other pathway recycled by-products to produce a single substance, such as fiber extraction [4,5]; production of cellular protein by microbial fermentation [6]; protein recovery [1,7]; biological hydrogen production [8,9]; preparation of methane from potato residues [10]; production of pullulan polysaccharide [11]; production of microbial flocculants [12,13], preparation the potato protease inhibitors (PPIs) from wastewater [14] are just a few. Most of these methods remain theoretical in the laboratory and have not completely solved the pollution problem or recovered by-products. In this case, the value of the converted product is lower than the investment cost, which greatly hinders the industrial application of many of these biotechnologies. In our preliminary work, microbial liquid fermentation conversion of potato starch by-products for cellular protein positively impacted COD removal from wastewater [1,6]. Based on that, the recovery biotechnology was optimized for industrial production, the recyclable and economically valuable substances in by-products were reclaimed more comprehensively and wastewater recycling benefitted pollution remediation. In the process of industrial recovery biotechnology of potato starch by-products, microbial fermentation performance impacted all downstream unit operations and by-product conversions [15], such as the degradation of cellulose, hemicellulose, and pectin, conversion of proteins. In addition, solid–liquid separation and energy consumption were also affected. Lowering moisture levels decreased energy consumption and represented a step in the right direction [16].

*Aspergillus niger* efficiently converts the complex structure of biomass resources by secreting large amounts of hydrolytic and oxidative enzymes [17,18], from which the relevant genes were identified [19], and is the most wildly used microbial strain in industry. Additionally, as a safe use for enzyme and metabolite production [20], *A. niger* has great potential industrial reuse of biological waste. Such as cellulase production [21], hemicellulase production [22] and pectinase production [23]. In addition, *A. niger* positively impacted on COD removal in sewage treatment [24]. In terms of bioflocculation, potato starch wastewater was utilized by *A. niger* to produce flocculants by optimizing production, the removal of COD and turbidity of potato starch wastewater reached 91.15% and 60.22%, respectively. These studies showed that *A. niger* played an indispensable role in biomass recycling and was a flocculating microorganism.

Flocculation by *A. niger* in the sedimentation process after fermentation was affected by a variety of factors and the flocculation mechanism in biological systems was intricate and many theories had been put forward on it: (1) Electric neutralization mechanism. The electric neutralization of flocculants occurred between flocculant molecules with opposite charges and flocculated particles. Due to the electrostatic attraction of flocculant molecules, the charge density between them and particles decreased, as do the repulsions between particles and flocculants, which eventually led to flocs formation [25]. (2) Adsorption bridging mechanism. The distance of the microbial flocculant extending from the particle surface to the solution exceeded particle repulsions, which resulted in adsorption bridging. The effectiveness of the bridging mechanism depended on the molecular weight, active group, charge on the molecule, hydrogen bonding of the flocculation system, van der Waals forces, and other microbial flocculant factors [26,27]. (3) Compression electric double layer mechanism. The flocculant was thought to act with colloidal particles via hydrogen bonds, van der Waals forces, electrostatic attractions, which resulted in the electric double layers between particles being compressed and overlapped, and the charges of colloidal particles were combined by flocculant molecules with opposite charges, which lowered the repulsion between particles and destabilized the colloidal system to form flocs [28,29]. (4) Sweeping effect. When the flocculant was added to the solution, sedimentation formed due to adsorption bridging or electric neutralization. The sedimentation swept or netted the colloidal particles in the solution during flocculation sinking and resulted in precipitation. (5) Chemical reaction mechanism. Flocculant molecules and colloidal particles were thought to react chemically to form large flocs that ultimately precipitate from the solution.

**Figure 1:**
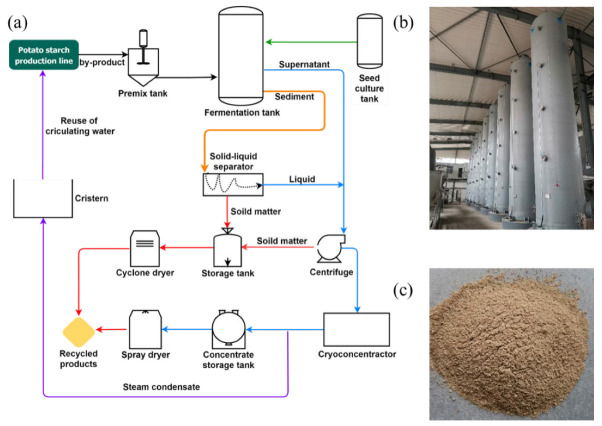
(**a**) Industrial reutilization biotechnology of potato starch by-products from a plant with an annual output of 1000 tons of starch and composed of three parts: Microbial fermentation production line, Solid-liquid separation production line, Drying and recovery production line. (**b**) Real photo of Microbial fermentation production line in the factory. (**c**) Recycled products.

In this study, we developed a practical industrial recovery for potato starch by-products by *A. niger* and fundamentally solved the wastewater pollution problems with a closed-loop cycle. The circulating water reduced the water consumption of the potato starch production line and thus reduces the production cost. The production process is as follows (Figure 1a): (1) All by-products (potato residues and wastewater) from the potato starch production line were transported to the Premix tank that maintained a by-products temperature of 30–35 °C by stirring and heat exchange, followed by transportation into the fermentation tank. Activated *A. niger* (from the Seed culture tank) was added into the fermentation tank (solid green line), followed by the air injection to start fermentation at a ventilation ratio of 0.01 VVM (air system not shown). (2) After fermentation (72 h), the solid-liquid stratification resulted in sedimentation (for 2 h); the supernatant was centrifuged for solid–liquid separation (solid blue line), and the solid was taken to a storage tank. The liquid was spray-dried after low-temperature (50 °C) concentration, and the solid was recovered (solid red line). (3) The sediment in the fermentation tank was discharged into the solid–liquid separator for preliminary solid–liquid separation (solid pale brown line), and the recovered solid matter was transported to the storage tank (solid red line). The liquid entered the water treatment unit of the supernatant for secondary solid-liquid separation. The solid substances in the storage tank were dried by the cyclone drying system to become recycled finished products and the dry matter content was about 92%, *w*/*w* (solid red line). Most of the water became a steam condensate during low temperature (50 °C) concentration and entered the potato starch production line for cleaning potatoes and starch (solid purple line). All units formed a closed-loop cycle.

*A. niger* was applied to convert by-products (potato residues and wastewater) into multifunctional potato powder. During production, solid-liquid separation was an important step during sedimentation process after fermentation, bioflocculation directly affected the subsequent production units by affecting the settling substance water content. Due to high-water levels of settling substances, the energy consumption and production costs of dehydration and drying increased [16]. The conversion effect directly determinized the value of the end product, multifunctional potato powder, which contained dietary fiber, proteins, vitamins, amino acids, etc. (data not shown), with applications in medical care, medical raw materials, and food additives. In addition, *A. niger* has been confirmed as a flocculation microorganism [30,31]. The possible flocculation mechanism was inferred by analyzing the soluble components with flocculation in the fermentation broth and the changes in the main components of potato starch by-products converted by *A. niger*, and by analyzing factors that affected the flocculation during sedimentation. It facilitates energy savings and cost reductions through subsequent fermentation optimization.

## 2. Materials and Methods

### 2.1. Strains and Cultural Conditions

Fungal strain *Aspergillus niger*, isolated and preserved in the Laboratory of Microbiology of Harbin Institute of Technology, was used in these experiments [6]. Spore culture medium: bran passed through 40 mesh sieve, mixed bran and water 4:6 ratio, sterilized at 121 °C for 30 min, then cultured at 30 °C for 5−7 d until *A. niger* reached 10^9^ spores g^−1^. The potato starch production line of the factory has an annual output of 1000 tons of starch, the by-products contain 3.5−4% dry matter. The liquid fermentation volume of each tank was 24 t/30 t with a 0.1% (*w*/*w*) inoculation amount of *A. niger* spores, cultured at 30 ± 2 °C for 72 h, and the ventilation ratio by 0.01 VVM (volume of air per volume of liquid per minute). The fermentation tank was an airlift fermentation tank developed independently by the Harbin Institute of Technology.

### 2.2. By-Products Component Analysis

Microbial fermentation decomposed and metabolized biomass [4]. Analyzing the changes of by-product components before and after fermentation intuitively verified the conversion effect of *A. niger* on by-products and provided mechanistic insights or factors that influence flocculation. Several experiments analyzed the primary by-product components before and after fermentation. Samples were taken after 24 h, 48 h, and 72 h, then layered for 2 h. The supernatant was used to determine COD levels, pH, and protein content. The precipitates were gathered and dried at 80 °C for 24 h, ground, and passed through 100 mesh sieves to determine cellulose, hemicellulose, and pectin levels.

### 2.3. Extraction and Characteristics of Flocculating Substance (FS)

All potato starch by-product samples were taken from Jilin MaoQuanShuBao Biotechnology Development Co., Ltd., (Changchun, China). FS was prepared by sampling after fermenting for 72 h. The fermentation solution was centrifuged at 5000 rpm 4 °C for 10 min, stirred with a double volume of pre-chilled 95% ethanol, stored at 4 °C for 24 h, then centrifuged at 5000 rpm for 10 min at 4 °C. The precipitates were collected and freeze-dried for FS.

A 1% solution of FS lyophilized powder was prepared for subsequent experiments. Components in FS were preliminarily analyzed by full band scanning. A 200 μL FS solution was added to 96 well plates and measured using a microplate reader (Infinite M200 Pro, Tecan, Männedorf, Switzerland) at wavelengths from 230–1000 nm. FTIR (Nicolet 6700, Thermo Fisher Scientific, Waltham, MA, USA) investigated FS functional groups. Gel Permeation Chromatography (GPC, Agilent 120, Walterbloom, Germany) determined the FS molecular weights (column model, Agilent PL aqua gel-OH 8 μm). The Monosaccharide composition analysis was carried out by Ion Chromatography (ICS5000, Thermo Fisher Scientific) with an electrochemical detector, Dionex™ CarboPac™ PA20 (150 × 3.0 mm, 10 μm, Thermo ScientificTM, Waltham, MA, USA) liquid chromatography column with a 5 µL injection volume. The GPC and monosaccharide composition tests were performed by shiyanjia lab (www.shiyanjia.com) (accessed on 21 January 2022).

### 2.4. FS Stability Analysis

The effects of different temperatures and initial pH levels were studied on a kaolin flocculation system to evaluate the stability of FS flocculation. Several experimental groups determined the stability of FS flocculation: a 0.5% (*v*/*v*) FS solution (1%) was heated at 30 °C, 40 °C, 50 °C, 60 °C, 70 °C, 80 °C, 90 °C and 100 °C for 0.5 h. After cooling, the 0.3% (*w*/*v*) kaolin suspension was flocculated, and the flocculation results were compared. The pH of the 0.3% (*w*/*v*) kaolin suspension was adjusted to 3, 4, 5, 6, 7, 8, 9, and 10. A 0.5% (*v*/*v*) FS solution (1%) was added to each group, stirred at 30 °C for 0.5 h, then stood for 0.5 h to compare the flocculation effect.

### 2.5. Analytical Methods and Statistical Analysis

Changes in kaolin flocculation morphologies by FS were observed, and all samples were characterized by SEM (QUANTA FEG 250, FEI, Hillsboro, OR, USA). Kaolin solid powder, FC powder, floc formed with 0.3% (*w*/*v*) kaolin solution, 0.08% (*w*/*v*) CaCl_2_ and 0.5% (*v*/*v*) FS solution (1%) were fixed with 2.5% glutaraldehyde for 12 h at 4 °C then dehydrated by different concentrations of ethyl alcohol. After drying, the surface state of FS and kaolin before and after FS flocculation were observed by SEM. The surface charges of kaolin, FS, and the flocculated kaolin treated by FS were determined based on zeta potential measurements. All samples were measured at pH 7/25 °C (Zeta-PALS, BIC, Shelton, CT, USA). Aliquots of 100 mL 2 mol·L^−1^ EDTA, 100 mL 0.5 mol·L^−1^ HCl, and 100 mL 5 mol·L^−1^ urea was added into the flocs to determine whether there was exclusive adsorption during floc formation in the ternary systems of kaolin, CaCl_2_, and FS, respectively, which was the sedimentation after supernatant removal in a system of total volume 100 mL, comprising 0.3% (*w*/*v*) kaolin solution, 0.08% (*w*/*v*) CaCl_2_, and 0.5% (*v*/*v*) FS solution (1%), pH 7. Changes in flocs were observed and recorded.

Cellulose and hemicellulose were determined by the Van Soest method [32]. Pectin was determined by the calcium pectate method [33]. COD was determined by the dichromate method [34]. The total sugar content of the FS was determined by the phenol sulfuric acid method [35]. The protein content of FS was determined by the Bradford method [36]. The flocculating rate was determined by the research method [37] and the flocculation system was substituted by 0.3% (*w*/*v*) kaolin solution, 0.08% (*w*/*v*) CaCl_2,_ and 0.5% (*v*/*v*) FS solution (1%). Three parallel experiments were conducted for all experimental groups and the data was analyzed using Microsoft Office Excel 2016 (Microsoft, Redmond, DC, USA).

## 3. Results and Discussion

### 3.1. Changes in By-Product Components

**Figure 2:**
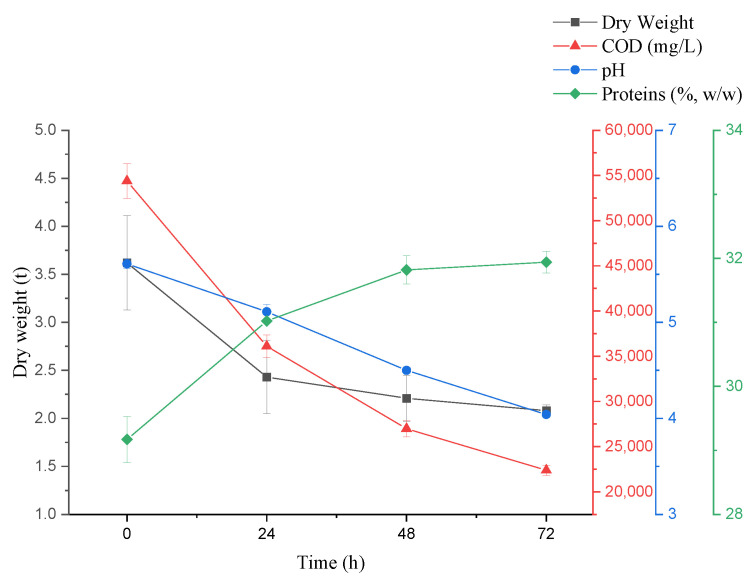
Component changes of wastewater composition. Error bars show a standard error from triplicate observations.

**Figure 3:**
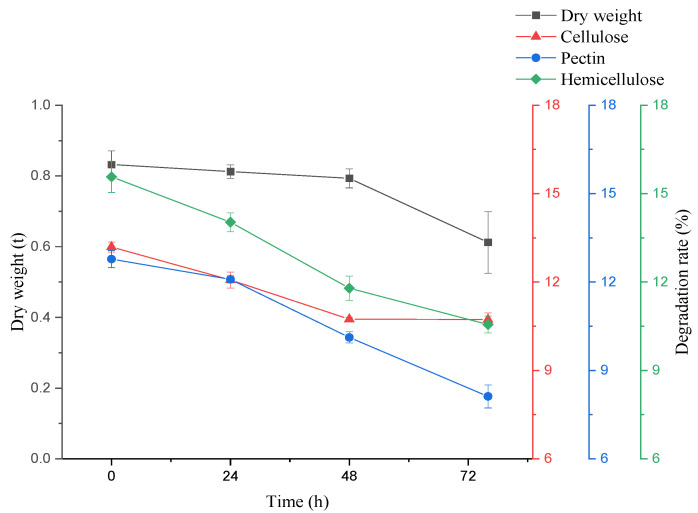
Component changes of sediment composition changes. Error bars show a standard error from triplicate observations.

**Figure 4:**
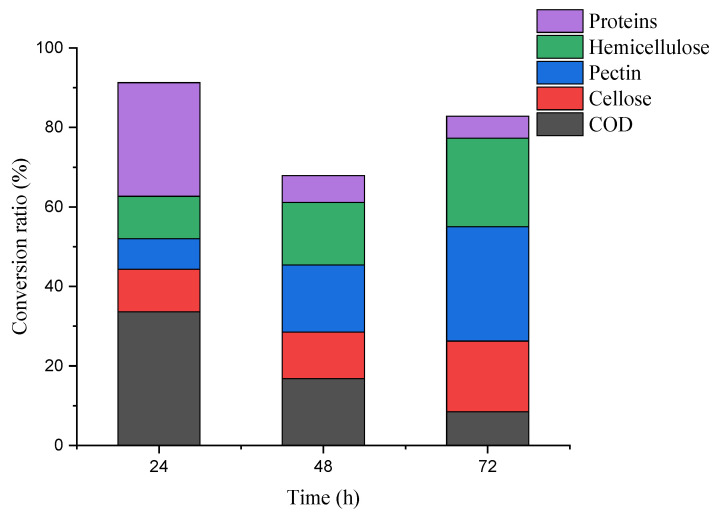
The conversion ratio of COD, cellulose, pectin, hemicellulose, and proteins per 24 h.

COD levels (Figure 2) decreased with fermentation time. After 72 h, the removal rate was 58.85%, lower than 65.92% of the previous 150 m^3^ volume fermentation by mixing microorganisms for 6 d [1] and much lower than anaerobic sludge (AS) and co-cultured microalgae [8]. Given that COD removal was related to flocculation [38,39], it indirectly helped investigate the correlation between the main components changes of by-products and flocculation in this process. Meanwhile, the pH decreased with time from 5.61 to 4.04. The protein contents in the supernatant decreased rapidly in the first 24 h to the decrease from 24–72 h, and the conversion efficiency was 37.09% after 72 h. The utilization of basic protein may have caused the pH decline [40] or the *A. niger* produced organic acids [41]. Generally, potato residues contain abundant celluloses, hemicellulose, and pectin [42], predominantly polymers with complex structures polymerized by various sugars. As shown in Figure 3, after 72 h of fermentation, the contents of cellulose, hemicellulose, and pectin decreased by 40.19%, 50.14%, and 53.29%, respectively. The conversion rates of cellulose and hemicellulose were lower than the previous study [1]. These polysaccharides decomposed into adequate monosaccharides (reducing sugar) by *A. niger* as carbon sources for growth. As shown in Figure 4, the removal ratio of COD in the first 24 h maximized at 32.62%, while the degradation ratio of cellulose, pectin, and hemicellulose occurred in the first initial 24 h (10.69%, 7.67%, and 10.96%, respectively), and conversion ratio of proteins maximized at 28.62%, which indicated that in the first 24 h, the COD removal in wastewater was mainly related to the protein conversion. In addition, the pH of wastewater decreased. This may be due to the basic proteins in wastewater conversion and utilization by *A. niger* [40]. Fermentation from 24 h to 48 h, the degradation ratio of cellulose was insignificant (11.72%), but the hemicellulose and pectin degradation ratio improved considerably (15.79% and 16.87%, respectively), and the conversion ratio of proteins reduced (6.71%). From 48 h to 72 h, the conversion ratio of hemicellulose and pectin improved considerably (22.30% and 28.75%, respectively), which may be due to the *A. niger* elevated viability of hemicellulase and pectinase, but the conversion ratio of cellulose improved slightly (17.79%), which may be due to glucose in a certain concentration inhibited cellulase [43]. The conversion ratio of proteins in the wastewater reduced (5.53%) and the pH decreased to 4.04. From 24 h to 72 h, the COD total removal ratio was at 25.24% and the total conversion ratio of cellulose, hemicellulose and pectin occurred (29.50%, 38.08%, and 45.62%, respectively), while the total conversion ratio of proteins was at 12.24%. This indicated that the COD removal from 24 h to 72 h was mainly related to the degradation of cellulose, hemicellulose, and pectin. This may be due to the degradation of three main polysaccharide components: cellulose degradation for providing glucose for *A. niger* growth priority utilization; pectin degradation converted to most of the monosaccharides may combine with unconverted proteins in the water; furthermore, the monosaccharides from hemicellulose degradation, such as arabinose and galactose were bound to proteins in the wastewater. Most of the monosaccharides after hemicellulose and pectin degradation were bound to the unconverted proteins and constituted the initial FS components. All the results indicated that in the production process, the proteins from wastewater provided a nitrogen source for *A. niger* growth, while the degradation of cellulose, hemicellulose, and pectin provided a carbon source. The COD removal was correlated with the conversion of cellulose, hemicellulose, pectin, and proteins by *A. niger*.

### 3.2. Composition and Functional Groups

**Figure 5:**
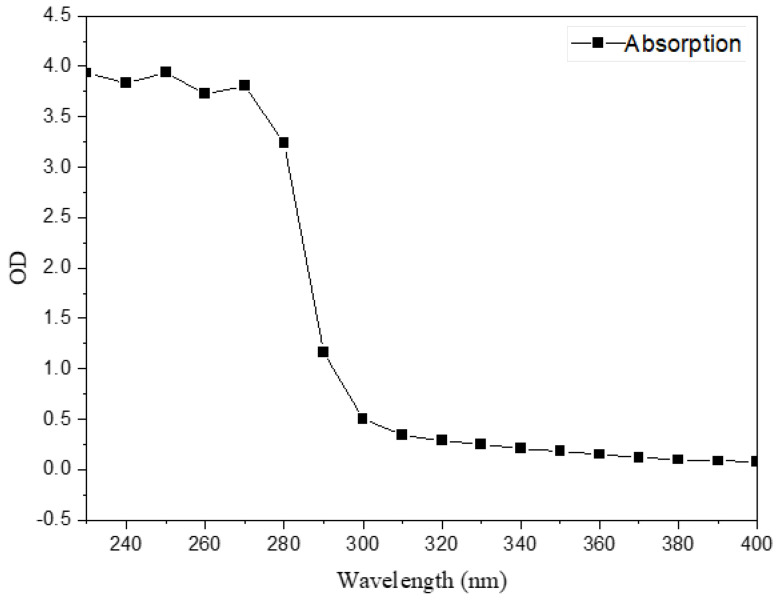
Full band scanning results of FS.

Full band scanning results demonstrated that a 1% FS solution had absorbed at 280 nm (Figure 5), which indicated that FS contained protein. By the determination of protein content, the existence of protein substance in FS was further verified, and the proportions of the protein content were determined to be 45.55% (*w*/*w*): the proportions of the polysaccharides content were determined to be 28.07% (*w*/*w*), the results indicated that the main components of FS were proteins and polysaccharides. Similar results have been found [44].

**Figure 6:**
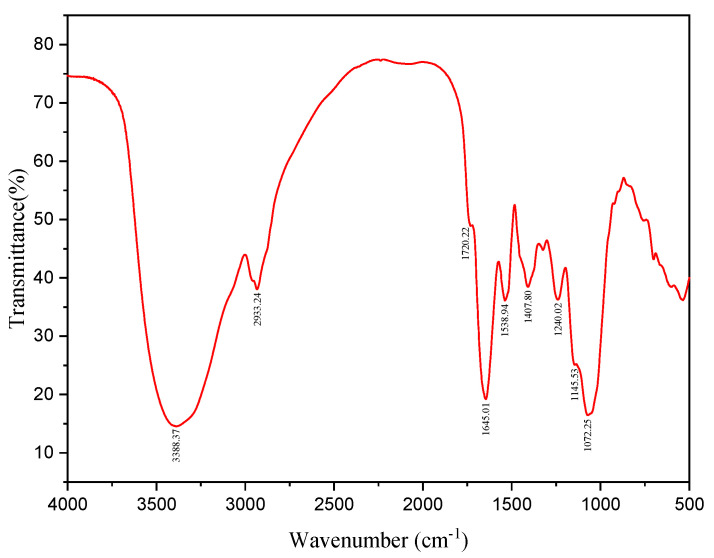
FTIR spectroscopy of FS.

The FTIR spectrum of FS (Figure 6) shows typically broad and sharp stretching vibrations between 3000–3700 cm^−1^, and the broad absorption wavelength at the peak of 3388.37 cm^−1^ was from -OH stretches (polysaccharides) and amino groups (proteins). The peak at 2933.85 cm^−1^ was due to asymmetric CH sugar stretches; the peak at 1720.22 cm^−1^ was characteristic of C=O stretching vibrations in -COOH, while the peak at 1645.01 cm^−1^ was due to C=O stretching vibrations caused by the protein amide bond, that confirmed the presence of carboxylates [37]. The peak at 1407.80 cm^−1^ was caused by variable angle C-H vibrations; the peaks at 1240.02 cm^−1^ and 1072.25 cm^−1^ were attributed to two kinds of C-O stretching vibrations—the C-O-C characteristic absorption peak of ether in polysaccharides ring, and a C-O-H absorption peak, characteristic of all sugar moieties [45]. The weak absorption at 927.61 cm^−1^ indicated the polysaccharides contained a β-Type glycosidic bond. Those results indicated the FS functional groups comprised hydroxyl, carbonyl, carboxyl groups, and amide groups, which have a high binding capacity in flocculation.

### 3.3. Molecular Weight and Monosaccharide Composition

**Figure 7:**
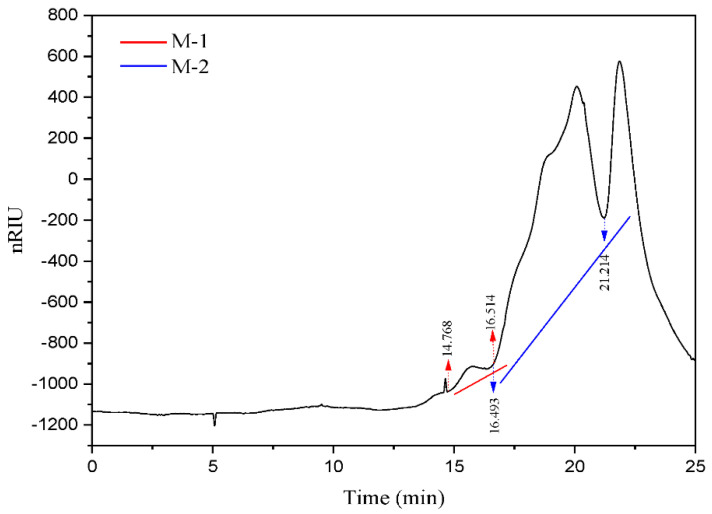
GPC raw data graph: the red line represents M-1 and the blue line represents M-2, which indicate the two molecular weight distributions of FS.

The FS molecular weight distributions were highly important, particularly when bridging was the primary flocculation mechanism [27]. GPC chromatography showed that FS had a bimodal molecular weight distribution (Figure 7). The average molecular weight (Mw), molecular mass (Mn), and polydispersity (PDI) results are shown in Table 1. The Mw, Mn, and PDI of M-1 were 7.3792 × 10^6^ Da, 7.2665 × 10^6^ Da, and 1.0156, respectively. The Mw, Mn, and PDI of M-2 were 1.7741 × 10^6^ Da, 1.4987 × 10^6^ Da, and 1.1838, respectively. The molecular weight distributions of the two polymers are shown in Figure S1. FTIR results showed protein functional groups in FS. In addition, full band scanning and protein content detection of FS also suggested the double peaks of GPC chromatography might be due to proteins and polymer compositions by polysaccharides and proteins. Given that the biopolymer molecular masses with flocculation activity generally exceed 0.102 × 10^6^ Da, flocculants with higher molecular masses involve more adsorption points for bridging and resulted in larger flocs [46,47]. The two molecular weight distributions of FS were considerably higher than 0.102 × 10^6^ Da, which provided the building blocks for bridging during flocculation. In addition, the flocculant with two molecular masses distributions demonstrated a better flocculation effect [48].

**Figure 8:**
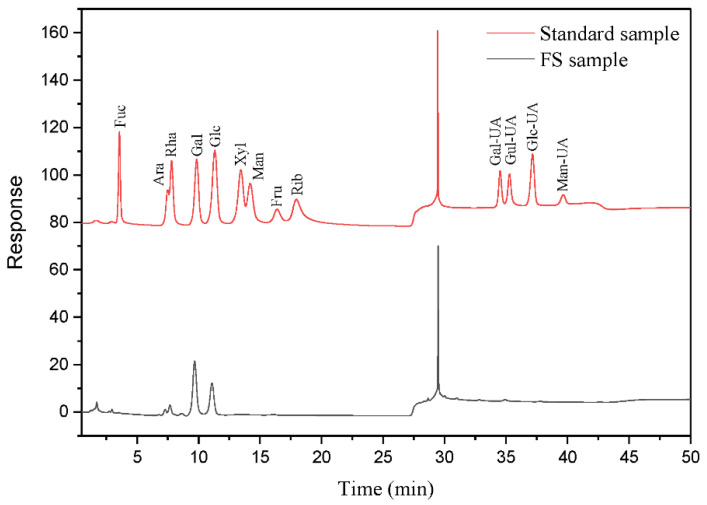
Monosaccharide ion chromatogram of polysaccharide: the red line represents mixed monosaccharides standard, and the black line represents the FS sample.

Monosaccharide analysis (Figure 8) showed that polysaccharide components in FS were composed of Ara, Rha, Gal, Glc, Xyl, Gal-UA, and Glc-UA (molar ratios—11.72:9.54:55.44:24.40:3.85:4.71:1.09, respectively). Table 1 shows, that the Gal-UA and Glc-UA content levels were 3.47% and 4.57% (*w*/*w*), respectively, which supply carboxyl groups to the FS molecular chain to produce flocculation in wastewaters [49]. Ara, Rha, and Gal as neutral sugar components in the RG-I domain of the pectin polymers [50], and Gal-UA as the main component of the HG domain are all components of potato pectin [51]. In addition, Ara and Gal are also the constituents of hemicellulose molecules [52]. The FS monosaccharide composition results indicated that potato residue hemicellulose and pectin degradation correlated with flocculation. This may explain that the COD removal was mainly associated with the conversion of hemicellulose and pectin during fermentation from 24 h to 72 h.

### 3.4. Stability Analysis of FS

**Figure 9:**
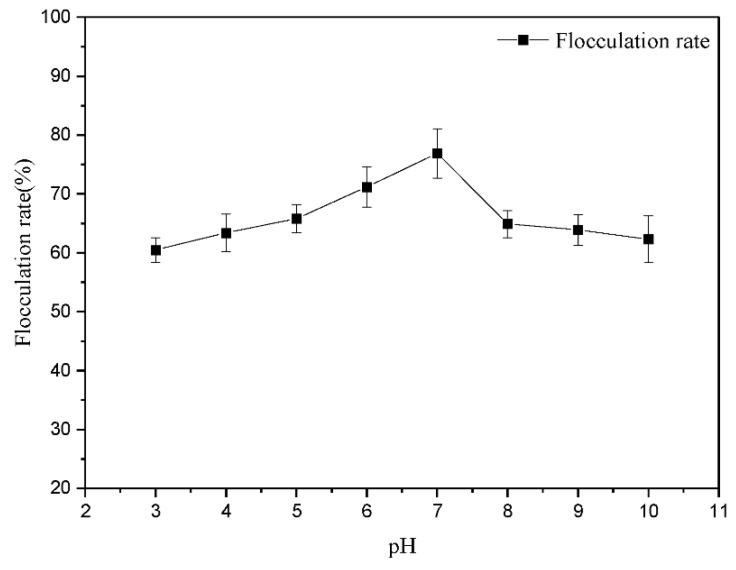
The effects of pH on the FS stability. Error bars show standard errors among triplicate observations.

Active ingredients of FS components affect flocculation, and pH and temperature may affect the stability of those components. By comparing the flocculation effects of FS on kaolin suspensions with different initial pH levels, the lowest flocculation rates (about 60%) occurred under strongly acidic or alkali conditions, but the highest flocculation rate (76.91 ± 4.21%) occurred at pH 7 (Figure 9). The maximum influence range by pH on the flocculation rate was approximately 20%, attributed to the weakening of FS spatial charge arrangements under different pH conditions [45]. This indicated that pH did not significantly affect the flocculation efficiency, which affected the electronic states of FS.

**Figure 10:**
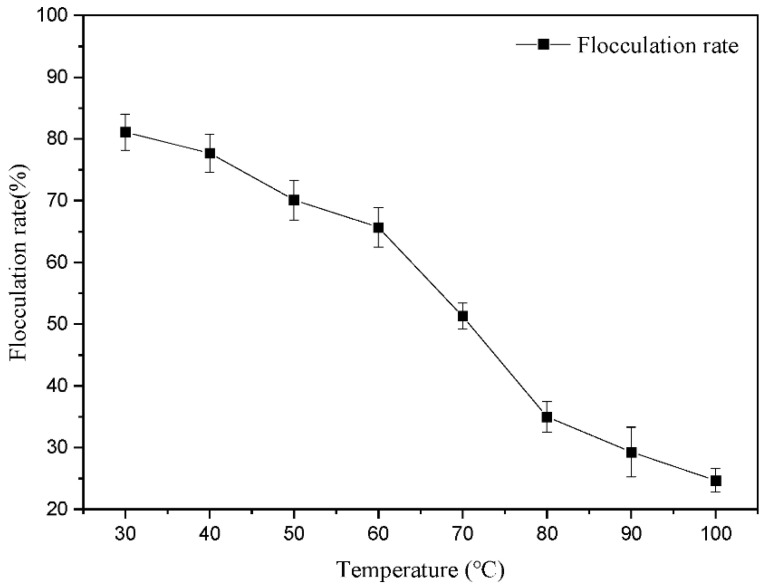
The effects of temperature on the FS stability. Error bars show standard errors among triplicate observations.

FS solutions were heated for 30 min at different temperatures, then cooled to room temperature to flocculate the kaolin suspension. The flocculation rate reached 81.10 ± 2.91% after heating at 30 °C and decreased at higher temperatures. The lowest flocculation rate occurred when FS was heated at 100 °C and decreased by 69.54%. These results indicated that temperature significantly impacted the flocculation of FS, which may stem from bond dissociation between proteins and polysaccharides or the disintegration of the flocculation system caused by protein structure changes at higher temperatures (Figure 10). A similar study demonstrated flocculants with protein as the main chain was sensitive to temperature [53]; this may explain the presence of proteins and polysaccharides in FS, which polymerize and adsorb particles to generate flocculation.

### 3.5. SEM, Zeta Potential, Inspection of Ionic and Hydrogen Bond

The kaolin particles observed by SEM were loose and unevenly shaped, while the flocculated kaolin particles gathered closely to form a large floc. Flocculated kaolin particles attached to the floc surface, which has the shape of some long chain structures similar to the bridging structure. All units formed a dense reticular structure and aggregated into larger flocs. The FS surface had a convex prism structure, which added more charge surface area and bridging sites during flocculation. The SEM results are shown in Figure S2.

**Figure 11:**
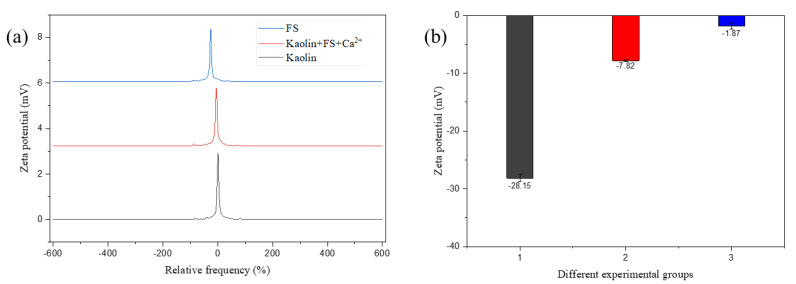
Zeta potentials of kaolin, kaolin + FS + Ca^2+^, and FS, respectively. Black solid lines and columns represent kaolin; red solid lines and columns represent kaolin + FS + Ca^2+^; and blue solid lines and columns represent the potential of FS, respectively. (**a**) Zeta potential frequency diagram (**b**) Zeta potential in different experimental groups.

The zeta potential of the kaolin suspension was −28.15 ± 0.56 mV at pH 7, which indicated a negatively charged kaolin particle surface. The solution zeta potential decreased to −7.82 ± 0.14 mV upon Ca^2+^ addition, likely due to the ionic neutralization which resulted in lower zeta potential. The FS zeta potential was −1.87 ± 0.46 mV at pH 7 (Figure 11), which indicated a negatively charged FS surface and possibly due to the existence of hydroxyl, carboxyl, and amide groups on FS. The change of kaolin potential before and after flocculation indicated a charge neutralization of FS occurred during flocculation. The pH stability results demonstrated the flocculation rate was not particularly sensitive to pH, indicating that charge neutralization may not be the main mechanism of flocculation.

Active functional groups on polymer FS form flocs by adsorption bridging with colloids or particles. Bonds between FS, kaolin, and Ca^2+^ were estimated by identifying the flocculated flocs with EDTA, HCl, and urea. As described in Table 2, flocs were so sensitive to EDTA and HCl that flocs disintegrated but were not sensitive to urea. Urea forms hydrogen bonds, while EDTA and HCl disrupted the ionic bonds between FS and particles and disintegrated flocs. This implied the binding mode between FS, kaolin, and Ca^2+^ was predominantly ionic bonds.

### 3.6. The Hypothesis of Flocculation Mechanism of A. niger during the Potato Starch By-Products Conversion

The functional group analysis results indicated the main functional groups in FS were hydroxyl, carboxyl, and amido groups; all provide adequate bridging sites [54]. SEM observations showed the FS surface had a prismatic structure and surface depressions, which provided additional surface area and bridging sites. The higher molecular weight provided more binding sites for bridging flocculants, which resulted in higher flocculation activities and larger flocs formation [54]. The two molecular weight distributions had better flocculation effects and more readily formed large flocs [48]. This indicated the primary flocculation by FS was bridging. However, the influence of pH on flocculation rate was limited and the change in flocculation rate was <20%. This indicated that charge adsorption was not the main flocculation mechanism. Differences in EPS polysaccharides secreted by microorganisms [55], indicated many FS polysaccharides came from potato starch by-product decomposition, especially hemicellulose and pectin. This indicated that the FS synthesis related to pectin and hemicellulose degradation and agreed with a previous study [44]. The sensitivity of FS to temperature indicated that glycoprotein in FS might drive flocculation. In practical sedimentation process, FS formed large flocs with particles via charge neutralization and adsorption bridges, and the large flocs attracted each other more easily to form a grid structure. SEM results demonstrated that the flocs were closely connected and formed a sweeping effect to reduce COD in the wastewater. The previous study showed that a bioflocculant (MBFA18) produced by fermentation of potato starch wastewater (juice) by *A. niger* was mainly polysaccharide [56]. Differently, *A. niger* was used to convert all the by-products simultaneously, and the flocculating substances (FS) generated were predominantly protein and polysaccharide in this study. The stability of FS is related to the different components of flocs. Flocs mainly containing protein are affected by temperature, while polysaccharide is relatively less affected, which is consistent with the results of the studies [12,13,44]. The factors affecting flocculation can be summarized as the active components of the flocculant, the products of different microorganisms and the raw materials, etc. Previous studies have shown that by using different sources of microorganism flocculants to floc potato starch wastewater, the predominantly flocculant mechanism was adsorption bridging.

## 4. Conclusions

**Figure 12:**
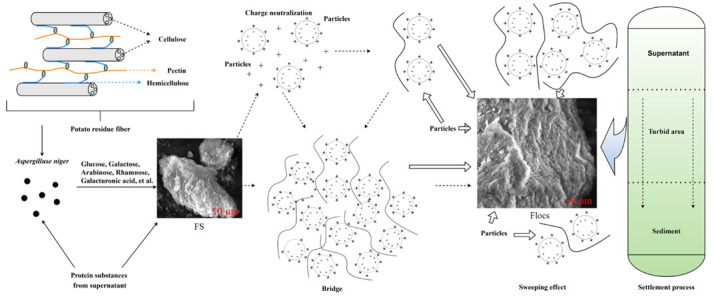
A hypothetical model for the flocculation by *A. niger* during industrial reutilization of potato starch by-products. The solid black arrows represent the conversion of potato starch by-products by *A. niger* and the formation of FS. The black dotted arrow represents the putative flocculation mechanistic process. The frame arrow represents the sweeping effect of large flocs by adsorption of surrounding matter.

This study developed a recovery biotechnology by *A. niger* to convert the potato starch by-products for industrial adaptation and solve the wastewater pollution completely. The impact of the COD removal, the protein conversion, and the degradation of by-products (cellulose, hemicellulose, and pectin) by *A. niger* was significant. Furthermore, flocculation by *A. niger* also played an important role in reducing the settling substance water content during production due to the solid-liquid separation affected the energy consumptions and the production costs. FS by *A. niger* primarily contained proteins and polysaccharides, with two molecular weight distributions (7.3792 × 10^6^ Da and 1.7741 × 10^6^ Da) and temperature sensibility. The monosaccharides that comprised the polysaccharides were predominantly composed of Gal, Glc, Ara, Rha, Gla-UA, Fru, and 72% of the monosaccharides were from hemicellulose and pectin conversion by *A. niger*. FS functional groups comprised hydroxyl, carbonyl, carboxyl groups, and amide groups. FS was negatively charged and connected with kaolin and Ca^2+^ by ionic bond and adsorption bridge, supplemented by charge neutralization to produce flocculation in a kaolin system. Combined with sedimentation during production, a sweeping effect on reducing COD may occur. The results indicated that flocculation by *A. niger* was mainly related to the conversion of proteins, hemicellulose, and pectin. Further research will examine the energy consumption of the production back-end unit and reduce the production costs by improving the conversion capacity of *A. niger* to proteins, hemicellulose, and pectin by optimizing fermentation to improve flocculation. In conclusion, a hypothetical model for the flocculation mechanism by *A. niger* during industrial reutilization of potato starch by-products is shown in the Figure 12.

Environmental pollution remains a severe problem in the potato starch industry, however, balancing investment costs and product value from practical biotechnologies with environmental pollution requires involved solutions. Compared with other potato starch by-product re-utilization directions, the cost considerations and pollution problems for practical industrial applications remain. Thus, this strategy of potato starch by-products merits serious consideration.

## Figures and Tables

**Table 1:** Composition and characteristics of FS.

Carbohydrate (%, *w*/*w*)	Proteins (%, *w*/*w*)	Molecular Weight Distributions				Monosaccharide Component (%, *w*/*w*)									
			Mw (g/mol)	Mn (g/mol)	PDI	Fuc	Ara	Rha	Gal	Glc	Xyl	Man	Fru	Gla-UA	Glc-UA
28.07 ± 0.12	45.55 ± 0.04	M-1	7.3792 × 10^6^	7.2665 × 10^6^	1.0156	0.42	8.80	8.69	49.94	21.98	0.51	0.54	3.47	4.57	1.06
		M-2	1.7741 × 10^6^	1.4987 × 10^6^	1.1838										

**Table 2:** Inspection of ionic and hydrogen bonds.

	EDTA	HCl	Urea
Phenomenon	The flocs disintegrated obviously; the supernatant was turbid	The flocs disintegrated obviously; the supernatant was turbid	The flocs no obvious disintegrated; the supernatant was clarified
Ionic bonds	+	+	−
Hydrogen bonds	−	−	+

“+” indicates the presence of a bond, and “−” indicates the absence of a bond.

## Data Availability

All individuals included in this section have consented to the acknowledgement.

## Supplementary Materials

The following supporting information can be downloaded at: https://www.mdpi.com/article/10.3390/microorganisms10091847/s1, Figure S1. The two molecular weight distributions. (a,c) represent the relationship between the elution volume and the detector response of M-1 and M-2, respectively; (b,d) represent the relationship between molar mass and *W* (*log M*) of M-1 and M-2, respectively. Figure S2. SEM observation of kaolin, kaolin after flocculation and FS. (a) kaolin; (b) kaolin after flocculation; (c) FS.

## References

[1] Liu B., Song J., Li Y., Niu J., Wang Z., Yang Q. (2013). Towards industrially feasible treatment of potato starch processing waste by mixed cultures. Appl. Biochem. Biotechnol..

[2] Wang R.M., Wang Y., Ma G.P., He Y.F., Zhao Y.Q. (2009). Efficiency of porous burnt-coke carrier on treatment of potato starch wastewater with an anaerobic–aerobic bioreactor. Chem. Eng. J..

[3] Hinken L., Huber M., Weichgrebe D., Rosenwinkel K.H. (2014). Modified ADM1 for modelling an UASB reactor laboratory plant treating starch wastewater and synthetic substrate load tests. Water Res..

[4] Cheng L., Zhang X., Hong Y., Li Z., Li C., Gu Z. (2017). Characterisation of Physicochemical and Functional Properties of Soluble Dietary Fibrefrom Potato Pulp Obtained by Enzyme-assisted Extraction. Int. J. Biol. Macromol..

[5] Gaudino E.C., Colletti A., Grillo G., Tabasso S., Cravotto G. (2020). Emerging Processing Technologies for the Recovery of Valuable Bioactive Compounds from Potato Peels. Foods.

[6] Liu B., Li Y., Song J., Zhang L., Dong J., Yang Q. (2014). Production of single-cell protein with two-step fermentation for treatment of potato starch processing waste. Cellulose.

[7] Waglay A., Karboune S., Alli I. (2014). Potato protein isolates: Recovery and characterization of their properties. Food Chem..

[8] Ren H.Y., Liu B.F., Kong F., Zhao L., Ren N. (2015). Hydrogen and lipid production from starch wastewater by co-culture of anaerobic sludge and oleaginous microalgae with simultaneous COD, nitrogen and phosphorus removal. Water Res..

[9] Singhvi M., Maharjan A., Thapa A., Jun H.-B., Kim B.S. (2021). Nanoparticle-associated single step hydrogen fermentation for the conversion of starch potato waste biomass by thermophilic *Parageobacillus thermoglucosidasius*. Bioresour. Technol..

[10] Parawira W., Murto M., Read J.S., Mattiasson B. (2007). A study of two-stage anaerobic digestion of solid potato waste using reactors under mesophilic and thermophilic conditions. Environ. Technol..

[11] Jin Z., Bai Y., Li X. (2021). Pullulanase Mutant. U.S. Patent.

[12] Guo J., Lau A.K., Zhang Y., Zhao J. (2015). Characterization and flocculation mechanism of a bioflocculant from potato starch wastewater. Appl. Microbiol. Biotechnol..

[13] Guo J., Zhang Y., Zhao J., Zhang Y., Xiao X., Wang B., Shu B. (2015). Characterization of a bioflocculant from potato starch wastewater and its application in sludge dewatering. Appl. Microbiol. Biotechnol..

[14] Liu Y.T., Hu X.P., Bai Y., Zhao Q.Y., Li T.P. (2020). Preparation and antioxidative stability of the potato protease inhibitors (PPIs) from potato starch waste-water. LWT Food Sci. Technol..

[15] Crater J.S., Lievense J.C. (2018). Scale-up of Industrial Microbial Processes. Fems Microbiol. Lett..

[16] Marcotte M., Grabowski S. (2008). Minimising energy consumption associated with drying, baking and evaporation. Handbook of Water and Energy Management in Food Processing.

[17] Cairns T.C., Barthel L., Meyer V. (2021). Something old, something new: Challenges and developments in Aspergillus niger biotechnology. Essays Biochem..

[18] Garrigues S., Kun R.S., Peng M., Bauer D., Keymanesh K., Lipzen A., Ng V., Grigoriev I.V., Vries R. (2022). Unraveling the regulation of sugar beet pulp utilization in the industrially relevant fungus Aspergillus niger. Iscience.

[19] De Vries R.P., Riley R., Wiebenga A., Aguilar-Osorio G., Amillis S., Uchima C.A., Anderluh G., Asadollahi M., Askin M., Barry K. (2017). Comparative genomics reveals high biological diversity and specific adaptations in the industrially and medically important fungal genus Aspergillus. Genome Biol..

[20] Frisvad J.C., Møller L., Larsen T.O., Kumar R., Arnau J. (2018). Safety of the fungal workhorses of industrial biotechnology: Update on the mycotoxin and secondary metabolite potential of Aspergillus niger, Aspergillus oryzae, and Trichoderma reesei. Appl. Microbiol. Biotechnol..

[21] Santos G.B., Filho L., Rodrigues J., Souza R. (2022). Cellulase production by Aspergillus niger using urban lignocellulosic waste as substrate: Evaluation of different cultivation strategies—ScienceDirect. J. Environ. Manag..

[22] Azzouz Z., Bettache A., Boucherba N., Eugenio L.D., Benallaoua S. (2020). Optimization of β-1,4-endoxylanase production by a new Aspergillus niger strain growing on wheat straw and application in xylooligosaccharides production. Authorea Prepr..

[23] Saa A., Moa B., Ana B., Aha B., Ama B., Mma A. (2022). Utilization of agro-industrial orange peel and sugar beet pulp wastes for fungal endo- polygalacturonase production—ScienceDirect. Saudi J. Biol. Sci..

[24] Chuppa-Tostain G., Tan M., Adelard L., Shum-Cheong-Sing A., François J.-M., Caro Y., Petit T. (2020). Evaluation of Filamentous Fungi and Yeasts for the Biodegradation of Sugarcane Distillery Wastewater. Microorganisms.

[25] Ajao V., Bruning H., Rijnaarts H., Temmink H. (2018). Natural flocculants from fresh and saline wastewater. Chem. Eng. J..

[26] Salehizadeh H., Shojaosadati S.A. (2001). Extracellular biopolymeric flocculants: Recent trends and biotechnological importance. Biotechnol. Adv..

[27] Wang L., Zhang B., Xiao J., Huang Q., Li C., Fu X. (2018). Physicochemical, functional, and biological properties of water-soluble polysaccharides from Rosa roxburghii Tratt fruit. Food Chem..

[28] Szewczuk-Karpisz K., Wisniewska M., Pac M., Choma A., Komaniecka I. (2014). Sinorhizobium meliloti 1021 Exopolysaccharide as a Flocculant Improving Chromium(III) Oxide Removal from Aqueous Solutions. Water Air Soil Pollut..

[29] Tang Y., Hu X., Cai J., Xi Z., Yang H. (2020). An enhanced coagulation using a starch-based coagulant assisted by polysilicic acid in treating simulated and real surface water. Chemosphere.

[30] Aljuboori A., Uemura Y., Osman N.B., Yusup S. (2014). Production of a bioflocculant from Aspergillus niger using palm oil mill effluent as carbon source. Bioresour. Technol..

[31] Nasir N.M., Yunos F., Jusoh H., Mohammad A., Lam S.S., Jusoh A. (2019). Subtopic: Advances in water and wastewater treatment harvesting of *Chlorella* sp. microalgae using Aspergillus niger as bio-flocculant for aquaculture wastewater treatment. J. Environ. Manag..

[32] Van Soest P.J., Robertson J.B., Lewis B.A. (1991). Methods for dietary fiber, neutral detergent fiber, and nonstarch polysaccharides in relation to animal nutrition. J. Dairy Sci..

[33] Emmett A.M., Carré M. (1926). A Modification of the Calcium Pectate Method for the Estimation of Pectin. Biochem. J..

[34] Clescerl L.S. (1998). Standard Methods for the Examination of Water and Wastewater.

[35] Masuko T., Minami A., Iwasaki N., Majima T., Nishimura S.I., Lee Y.C. (2005). Carbohydrate analysis by a phenol–sulfuric acid method in microplate format. Anal. Biochem..

[36] Walker J.M. (2002). Protein Protocols Handbook, The Volume 0||The Bradford Method for Protein Quantitation.

[37] Pu S., Ma H., Deng D., Xue S., Zhu R., Zhou Y., Xiong X. (2018). Isolation, identification, and characterization of an Aspergillus niger bioflocculant-producing strain using potato starch wastewater as nutrilite and its application. PLoS ONE.

[38] Rahman A., Meerburg F.A., Ravadagundhi S., Wett B., Jimenez J., Bott C., Al-Omari A., Riffat R., Murthy S., De Clippeleir H. (2016). Bioflocculation management through high-rate contact-stabilization: A promising technology to recover organic carbon from low-strength wastewater. Water Res..

[39] Tan S., Cui C., Chen X., Li W. (2016). Effect of bioflocculation on fouling-related biofoulants in a membrane bioreactor during saline wastewater treatments. Bioresour. Technol..

[40] Li Q., Ray C.S., Callow N.V., Loman A.A., Islam S., Ju L.K. (2020). Aspergillus niger production of pectinase and α-galactosidase for enzymatic soy processing—ScienceDirect. Enzym. Microb. Technol..

[41] Steiger M.G., Rassinger A., Mattanovich D., Sauer M. (2019). Engineering of the citrate exporter protein enables high citric acid production in Aspergillus niger. Metab. Eng..

[42] Meyer A.S., Dam B.P., Lærke H.N. (2009). Enzymatic solubilization of a pectinaceous dietary fiber fraction from potato pulp: Optimization of the fiber extraction process. Biochem. Eng. J..

[43] Hsieh C., Cannella D., Jørgensen H., Felby C., Thygesen L.G. (2014). Cellulase Inhibition by High Concentrations of Monosaccharides. J. Agric. Food Chem..

[44] Qi Z., Zhu Y., Guo H., Wang X., Shao Q. (2019). Production of Glycoprotein Bioflocculant from Untreated Rice Straw by a CAZyme-rich Bacterium, *Pseudomonas* sp. HP2. J. Biotechnol..

[45] Busisiwe M., Kunle O., Ncedo N., Uchechukwu N., Ezekiel G., Leonard M., Anthony O. (2016). Assessment of Bacillus pumilus Isolated from Fresh Water Milieu for Bioflocculant Production. Appl. Ences.

[46] Sobeck D.C., Higgins M.J. (2002). Examination of three theories for mechanisms of cation-induced bioflocculation. Water Res..

[47] Allen M.S., Welch K.T., Prebyl B.S., Baker D.C., Meyers A.J., Sayler G.S. (2004). Analysis and glycosyl composition of the exopolysaccharide isolated from the floc-forming wastewater bacterium *Thauera* sp. MZ1T. Environ. Microbiol..

[48] Gregory J., Barany S. (2011). Adsorption and flocculation by polymers and polymer mixtures. Adv. Colloid Interface Sci..

[49] Cosa S., Mabinya L.V., Olaniran A.O., Okoh A.I. (2012). Production and characterization of bioflocculant produced by *Halobacillus* sp. Mvuyo isolated from bottom sediment of Algoa Bay. Environ. Technol..

[50] Khodaei N., Karboune S. (2013). Extraction and structural characterisation of rhamnogalacturonan I-type pectic polysaccharides from potato cell wall. Food Chem..

[51] Yang J.S., Mu T.H., Ma M.M. (2018). Extraction, structure, and emulsifying properties of pectin from potato pulp. Food Chem..

[52] Larsen N., de Souza C.B., Krych L., Kot W., Leser T.D., Sorensen O.B., Blennow A., Venema K., Jespersen L. (2019). Effect of potato fiber on survival of Lactobacillus species at simulated gastric conditions and composition of the gut microbiota in vitro. Food Res. Int..

[53] Guo J., Yu J., Xin X., Zou C., Cheng Q., Yang H., Nengzi L. (2015). Characterization and flocculation mechanism of a bioflocculant from hydrolyzate of rice stover. Bioresour. Technol. Biomass Bioenergy Biowastes Convers. Technol. Biotransform. Prod. Technol..

[54] Yuan S.J., Sun M., Sheng G.P., Li Y., Li W.W., Yao R.S., Yu H.Q. (2011). Identification of key constituents and structure of the extracellular polymeric substances excreted by Bacillus megaterium TF10 for their flocculation capacity. Environ. Sci. Technol..

[55] Mx A., Han Z.A., Ca A., Lan H.A., Li S., Rya B., Yla B., Miao C., Jla B., Xwa B. (2021). A novel polysaccharides-based bioflocculant produced by Bacillus subtilis ZHX3 and its application in the treatment of multiple pollutants. Chemosphere.

[56] Byrne B.J., Geberhiwot T., Barshop B.A., Barohn R., Hughes D., Bratkovic D., Desnuelle C., Laforet P., Mengel E., Roberts M. (2017). A study on the safety and efficacy of reveglucosidase alfa in patients with late-onset Pompe disease. Orphanet J. Rare Dis..

