# Supplementary material for: Application of Aspergillus niger in Practical Biotechnology of Industrial Recovery of Potato Starch By-Products and Its Flocculation Characteristics
Source: Microorganisms. 2022 Sep 15;10(9):1847. doi: 10.3390/microorganisms10091847 (PMC9505473; doi:10.3390/microorganisms10091847)
Supplement: Supplementary file 1 [file microorganisms-10-01847-s001.zip › microorganisms-1925247-supplementary.pdf]

microorganisms MDPI

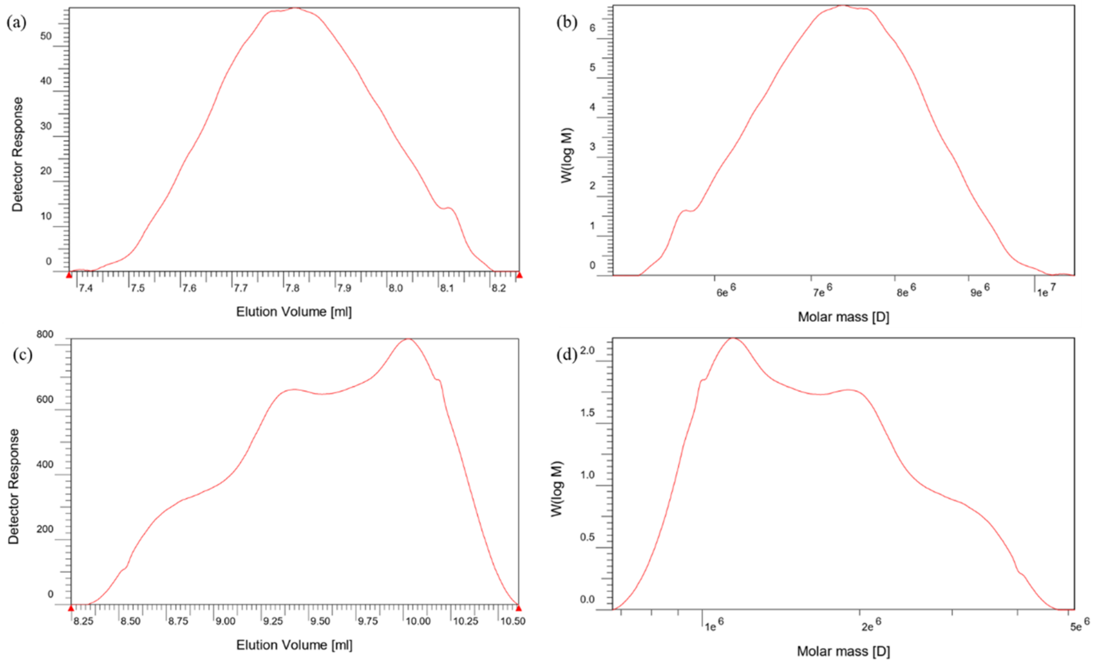

Figure S1. The two molecular weight distributions. (a) and (c) represent the relationship between the elution volume and the detector response of M-1 and M-2, respectively; (b) and (d) represent the relationship between molar mass and *W* (*log M*) of M-1 and M-2, respectively.

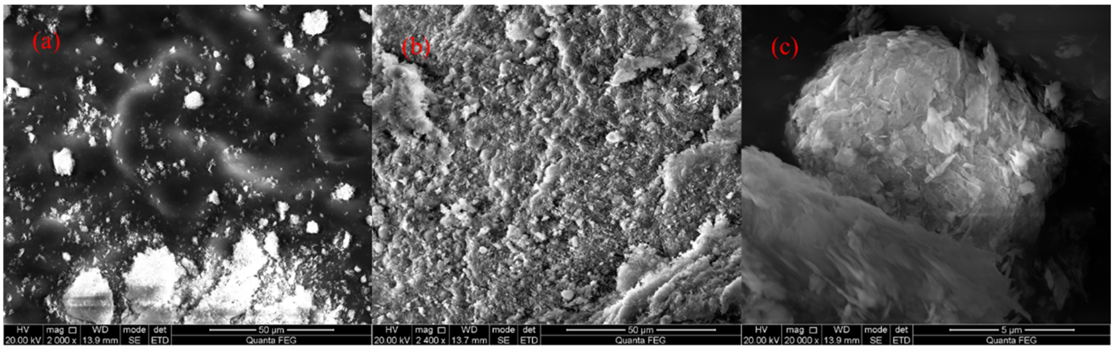

Figure S2. SEM observation of kaolin, kaolin after flocculation and FS. (a) kaolin; (b) kaolin after flocculation; (c) FS.
